# A Critical Appraisal of Evidence- and Consensus-Based Guidelines for Actinic Keratosis

**DOI:** 10.3390/curroncol28010093

**Published:** 2021-02-19

**Authors:** Anja Wessely, Theresa Steeb, Franz Heppt, Annkathrin Hornung, Matthias D. Kaufmann, Elias A. T. Koch, Frédéric Toussaint, Michael Erdmann, Carola Berking, Markus V. Heppt

**Affiliations:** 1 Department of Dermatology, Deutsches Zentrum Immuntherapie (DZI), Universitätsklinikum Erlangen, Friedrich-Alexander-University Erlangen-Nürnberg (FAU), 91054 Erlangen, Germany; anja.wessely@uk-erlangen.de (A.W.); theresa.steeb@uk-erlangen.de (T.S.); Heppt@psorisol.de (F.H.); annkathrin.hornung@uk-erlangen.de (A.H.); matthias.kaufmann@uk-erlangen.de (M.D.K.); elias.koch@uk-erlangen.de (E.A.T.K.); frederic.toussaint@uk-erlangen.de (F.T.); michael.erdmann@uk-erlangen.de (M.E.); carola.berking@uk-erlangen.de (C.B.); 2 Comprehensive Cancer Center Erlangen-European Metropolitan Area of Nuremberg (CCC ER-EMN), 91054 Erlangen, Germany

**Keywords:** actinic keratosis, solar keratosis, AGREE, level of evidence, practice guideline

## Abstract

Actinic keratoses (AK) are common lesions of the skin that can be effectively treated with several lesion- and field-directed treatments. Clinical practice guidelines assist physicians in choosing the appropriate treatment options for their patients. Here, we aimed to systematically identify and evaluate the methodological quality of currently available guidelines for AK. Guidelines published within the last 5 years were identified in a systematic search of guideline databases, Medline and Embase. Then, six independent reviewers evaluated the methodological quality using the tools “Appraisal of Guidelines for Research and Evaluation” (AGREE II) and “Recommendation EXcellence” (AGREE-REX). The Kruskal–Wallis (H) test was used to explore differences among subgroups and Spearman’s correlation to examine the relationship between individual domains. Three guidelines developed by consortia from Canada, Germany and the United Kingdom were eligible for the evaluation. The German guideline achieved the highest scores, fulfilling 65 to 92% of the criteria in AGREE II and 67 to 84% in AGREE-REX, whereas the Canadian guideline scored 31 to 71% of the criteria in AGREE II and 33 to 46% in AGREE-REX. The domains “stakeholder involvement“ and “values and preferences“ were identified as methodological weaknesses requiring particular attention and improvement in future guideline efforts.

## 1. Introduction

Actinic keratoses (AK) are common lesions of chronically sun-damaged keratinocytes in the epidermis, the upper layer of the skin [1]. They are most commonly found in areas that have been chronically exposed to ultraviolet (UV) radiation such as the head, face and the dorsal hands [2]. Affected areas usually present as red, scaly plaques with a rough surface [2]. AK can further progress to invasive squamous cell carcinoma of the skin [3]. However, it is currently not possible to predict which AK may progress and which do not, thus, consequent treatment of AK is recommended by several international practical guidelines, especially in high-risk patients [4,5,6].

Over the last decades, a broad variety of options have been licensed for AK treatment. They include lesion-directed options as cryotherapy and fractional laser therapy, which are suitable to treat single AK lesions. Another approach comprises field-directed treatments, which are usually deployed to treat larger areas of sun-damaged skin, for instance photodynamic therapy (PDT), which may be also effective in other skin diseases such as acne vulgaris, as recently shown by Del Duca et al. [7], microneedling and application of various topicals. Additionally, monotherapies can be combined for a stronger effect to achieve better results especially in difficult-to-treat or therapy-resistant AK patients [8,9,10,11,12]. The vast number of available and approved therapies may be both a blessing and a curse. Thus, up-to-date medical practical guidelines are valuable tools that help to select the most suitable and evidence-based approach for the individual patient with additional consideration of their personal preferences [13]. However, the provided recommendations should be developed in a structured process based on a sound methodological quality to ensure reliability and engagement.

In this study, we aimed to assess the methodological strengths and weaknesses of all currently available international guidelines on AK treatment using the two assessment instruments “Appraisal of Guidelines for Research and Evaluation (AGREE) II” and “AGREE-REX: Recommendation EXcellence” [14,15]. The widely used AGREE II appraisal tool from 2009 is an updated version of AGREE that was originally released in 2001. AGREE II consists of 23 items that are grouped in the six quality domains “scope and purpose”, “stakeholder involvement”, “rigor of development”, “clarity of presentation”, “applicability” and “editorial independence” as well as two items assessing the overall quality of the respective guideline. Recently, the AGREE-REX instrument was launched to complement guideline evaluation with AGREE II. AGREE-REX covers the topics clinical credibility and implementability and also assesses how values and preferences of target users, patients, policy and the guideline developers themselves have influenced the development of the recommendations.

## 2. Materials and Methods

### 2.1. Eligibility Criteria

In our appraisal, we only included guidelines developed by national or international consortia focusing on more than one option for the management of AK. They had to be published within the last 5 years, i.e., 2015–2019, as we only wanted to evaluate the most up-to-date guidelines. Furthermore, only English or German publications were included. Guidelines that had already expired or were not developed based on a systematic literature search followed by a structured consensus process, e.g., expert consent-based guidelines, were excluded.

### 2.2. Search Strategy and Selection of Guidelines

In order to identify potential guidelines for the evaluation, we systematically searched several guideline databases as well as Medline and Embase (both via Ovid) until 23 October 2019. The search included the terms “actinic keratosis/keratoses”, “solar keratoses”, “field cancerization”, “senile keratoses” and “precancerous lesions”. Besides, cross-references of included guidelines were screened as well. The detailed search strategies are shown in Supplementary Tables S1 and S2. The search results were screened for double hits. After their elimination, the remaining titles, abstracts or editorials were screened by two authors (M.V.H., T.S.), if they met the predefined eligibility criteria. Full-text guidelines of potentially relevant records were obtained and checked for eligibility again.

### 2.3. Data Extraction and Rating of the Guidelines

Background information including title, consortia and/or authors, country of origin, publication date, methodological approach and scope of all eligible guidelines were collected. Then, six independent reviewers (A.W., F.H., A.H., M.K., E.K., F.T.) evaluated their methodological quality using AGREE II and AGREE-REX as described previously [16]. Using AGREE II, the quality of each of the 23 items was assessed on a 7-point scale ranging from 1 (”strongly disagree”) to 7 (“strongly agree”) and similarly, the guideline’s overall quality was evaluated on a 7-point scale ranging from lowest to highest possible quality. Furthermore, the question “I would recommend this guideline for use” was answered by each reviewer with “yes”, “yes, with modifications” or “no”. The 9 items supplied by AGREE-REX were also assessed on a 7-point scale ranging from 1 (“lowest quality”) to 7 (“highest quality”) as described previously [16]. All evaluations using AGREE II and AGREE-REX were blinded towards the other evaluators’ assessments and performed independently. The platform “My AGREE Plus” provided by the AGREE consortium on https://www.agreetrust.org/ (last access: 31 May 2020) was used for evaluating the guidelines with the AGREE II instrument, whereas internally piloted data extraction spreadsheets (Microsoft Excel 2010) were used for the evaluation with AGREE-REX.

### 2.4. Analysis

Scores were calculated for each domain according to the instructions provided in the AGREE II and AGREE-REX instrument user manuals [15,17]. Total scores were expressed as percentages ranging from 0% as the worst to 100% as the best possible evaluation for each domain. Mean ± standard deviation (SD) was calculated for descriptive analyses. The Kruskal–Wallis (H) test was used to explore differences among subgroups and Spearman’s correlation to examine the relationship between individual domains and items of the instruments. *p*-values < 0.05 were considered statistically significant. Ratings were grouped in the three categories “strongly agree” (6 and 7 points), “partly agree” (3 to 5 points) and “strongly disagree” (1 and 2 points) and Fleiss’ Kappa was calculated in order to assess the interrater agreement of the six reviewers [18]. SPSS Statistics (version 24, IBM Corporation, Armonk, NY, USA) was used for all statistical analyses.

## 3. Results

### 3.1. Guideline Identification

**Figure 1:**
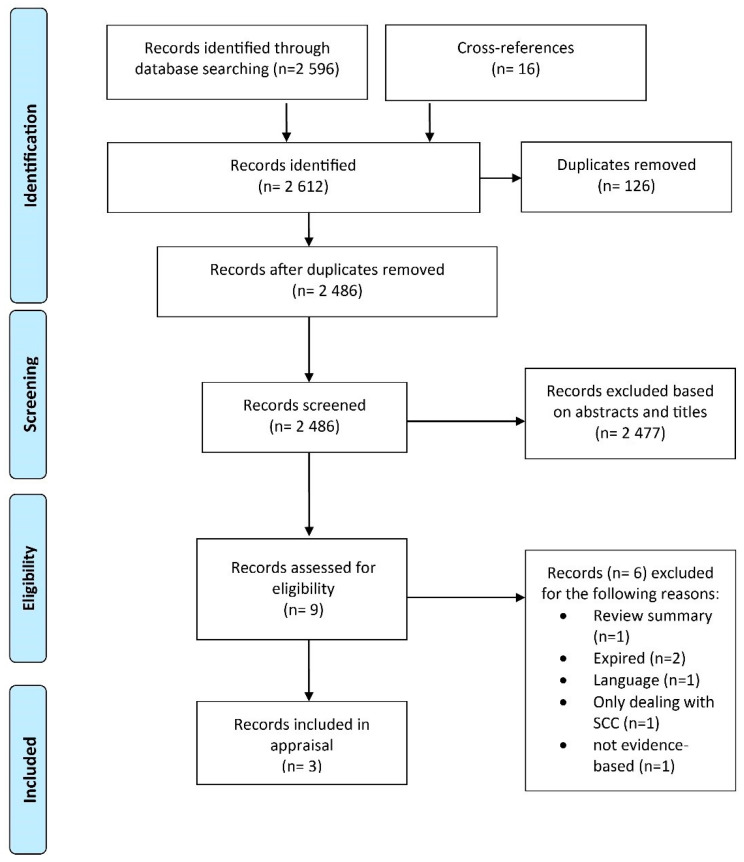
Flow chart of the guideline identification process according to the Preferred Reporting Items for Systematic Reviews and Meta-Analyses (PRISMA) guideline.

We initially identified 2612 records when searching the databases (Figure 1). After the elimination of double hits (*n* = 126) and title and abstract screening, nine records remained for full-text review. Six records were excluded, as they had already expired (*n* = 2) [19,20], only dealt with cutaneous squamous cell carcinoma (*n* = 1) [21], were not evidence-based (*n* = 1) [22] or did not meet our language eligibility (*n* = 1) [23]. Another record was dismissed, as it was a review summary and not a guideline [24]. Finally, the following three guidelines met our eligibility criteria and, therefore, were included in our assessment: the guideline of the Canadian Non-Melanoma Skin Cancer Guidelines Committee [4], the guideline of the British Association of Dermatologists from the United Kingdom (UK) [6] and the guideline developed by the Association of the Scientific Medical Societies in Germany (AWMF) and the German Cancer Society (DKG) [5,10,25]. The full-length German guideline is also available in English in the “Supporting Information” section of the short version [10].

### 3.2. Evaluation of the Guidelines

The interrater agreement of the six reviewers regarding AGREE II and AGREE-REX was rated as fair with a Fleiss’ Kappa of 0.299 (95% CI 0.263–0.336).

### 3.3. AGREE II

#### 3.3.1. Scope and Purpose

**Figure 2:**
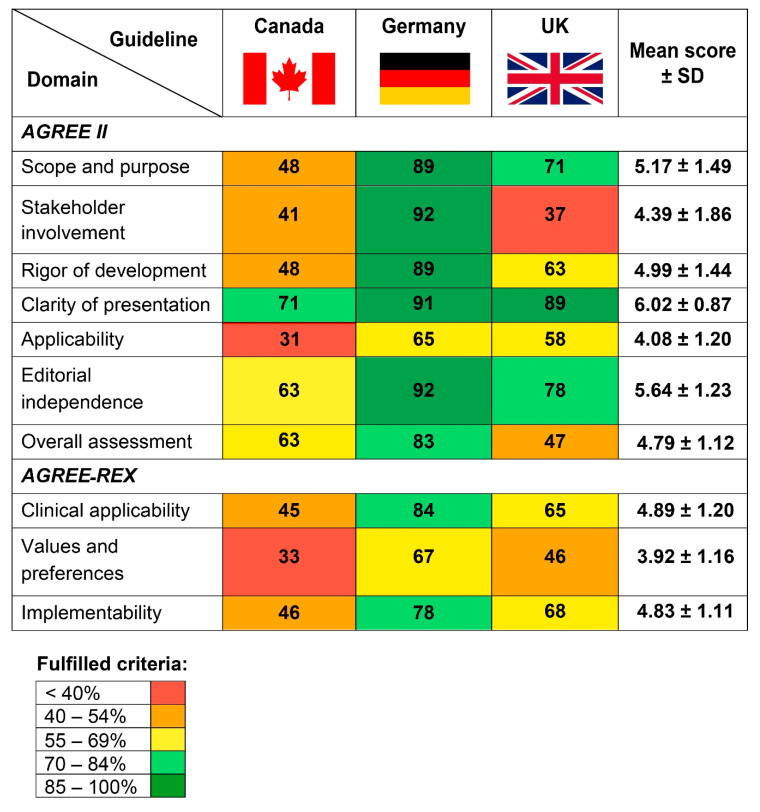
Heat map illustrating “Appraisal of Guidelines for Research and Evaluation” (AGREE II) and “Recommendation EXcellence” (AGREE-REX) scores of the three actinic keratoses (AK) guidelines evaluated by six independent reviewers.

This domain evaluates whether the main objectives of the guideline and the population for whom it was developed are clearly described. The average score was 5.17 (±1.49, Figure 2). The German guideline achieved the highest score fulfilling 89% of the criteria of this domain. The Canadian guideline was rated lowest with 48% and the UK guideline was rated in between achieving 71%. The German and the Canadian guideline significantly differed from each other (*p* = 0.01).

#### 3.3.2. Stakeholder Involvement

This domain covers the topics involvement of appropriate stakeholders and whether the views of the users that should deploy the guideline are represented. The average score was 4.39 (±1.86). The German guideline achieved a very high value of 92%, while the UK and Canadian guidelines achieved only 37 and 41%, respectively. The German guideline significantly differed from both the Canadian (*p* = 0.012) and the UK guideline (*p* = 0.009).

#### 3.3.3. Rigor of Development

The methodological approaches including a systematic and transparent identification of evidence are covered by the items of this domain. The mean score was 4.99 (±1.44). Again, the German guideline was rated as the one with the best methodological quality achieving 89% whereas the UK and the Canadian guidelines were rated worse with 63 and 48%, respectively. The German guideline also significantly differed from the Canadian guideline in this domain (*p* = 0.006).

#### 3.3.4. Clarity and Presentation

This domain evaluates the presentation of the provided recommendations including the clarity of recommendations or if key recommendations can be easily found in the guideline text at a glance. The mean score was 6.02 (±0.87). Both the German and the UK guideline fulfilled almost all criteria (89 and 91%, respectively) and also the Canadian guideline fulfilled more than 2/3 of the criteria (71%).

#### 3.3.5. Applicability

Processes concerning guideline implementation are evaluated in this domain. The mean score was 4.08 (±1.20). The German and UK guidelines achieved similar results (65 and 58%), while the Canadian guideline achieved the lowest rates fulfilling only 31% of the criteria. The German and Canadian guidelines also significantly differed in this domain (*p* = 0.004).

#### 3.3.6. Editorial Independence

The role of funding and competing interest of the experts that were involved in the development process is evaluated in this domain. The mean score was 5.64 (± 1.23). Again, the German guideline was rated as the best (92%) followed by the UK (78%) and Canadian guideline (63%). Similar to the other domains, the Canadian and the German guidelines were rated to be significantly different of each other (*p* = 0.035).

#### 3.3.7. Overall Assessment

This domain evaluates the overall quality and whether the reviewer would recommend to use the guideline in practice. The mean score was 4.79 (±1.12), and the German guideline was rated as the one with the best overall quality (83%). The UK and Canadian guidelines were rated lower with 63 and 47%, respectively. All reviewers recommended to use the German guideline without any modifications. The use of the UK guideline was also recommended, but half of the reviewers rated to use it with modifications. In contrast, the ratings regarding the recommendation to use the Canadian guideline were ambiguous: two reviewers rated to use it, two to use it with modifications while another two reviewers recommended not to use this guideline.

### 3.4. AGREE-REX

#### 3.4.1. Clinical Applicability

This domain assesses whether the recommendations were developed based on a thorough review of the existing literature and whether they are applicable for the intended users (e.g., physicians, patients). The mean score was 4.89 (±1.20). The fulfilled criteria in this domain ranged from 45% (Canada) and 65% (UK) to 84% (Germany). In this domain, the German and the Canadian guidelines significantly differed from each other (*p* = 0.002).

#### 3.4.2. Values and Preferences

This domain evaluates whether the preferences of the intended users, patients, policy/decision-makers and guideline developers have been taken into consideration during the guideline development process. The mean score was 3.92 (±1.16). The German guideline achieved 67%, whereas the UK and the Canadian guidelines were rated similar (46 and 33%, respectively). Here, the German and Canadian guidelines significantly differed again (*p* = 0.004). 

#### 3.4.3. Implementability

This domain asks how suitable the recommendations are for the patients and/or the health care system in which they should be implemented. The mean score of this domain was 4.83 (±1.11). The German guideline achieved the highest rates with 78%. The UK guideline was rated lower with 68%, and the Canadian guideline was rated with the lowest scores (46%). The German and Canadian guidelines also significantly differed in this domain (*p* = 0.004).

### 3.5. Correlations of the AGREE II and AGREE-REX Domains

**Figure 3:**
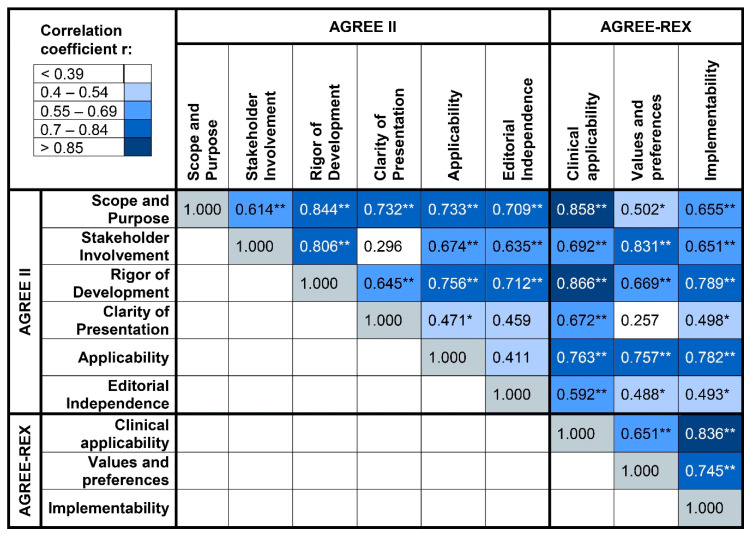
Correlations among the AGREE II and AGREE-REX domains. *: *p* < 0.05, **: *p* < 0.01.

Most of the AGREE II and the AGREE-REX domains were significantly positively correlated with each other (Figure 3). The domain “scope and purpose” was highly positively correlated with the domains “rigor of development” (r = 0.84) and “clinical applicability” of the AGREE-REX tool (r = 0.86). Additionally, the domain “stakeholder involvement” was highly correlated with the domains “rigor of development” (r = 0.81) and “values and preferences” (r = 0.83). Furthermore, the domains “clinical applicability” and “implementability” showed a high positive correlation (r = 0.84).

## 4. Discussion

AK are one of the most commonly diagnosed conditions in dermatology [26]. Due to the overwhelming number of available treatment options, choosing the most appropriate intervention for each patient can be challenging. In this study, we evaluated currently available guidelines on AK using the appraisal instruments AGREE II and AGREE-REX. The AGREE II tool and its previous version AGREE have already been successfully used in other evaluations in the field of dermatology and guideline development [27,28]. In an evaluation of published guidelines by the European Dermatology Forum (EDF), the assessment with AGREE highlighted that evidence- and consensus-based guidelines (“S3 level”) generally received the highest score in comparison to guidelines derived through either a structured consensus process, a systematic literature assessment or on informal consensus only [27]. Thus, identifying evidence- and consensus-based guidelines and their methodological strengths and weaknesses is essential for improving the overall quality of national as well as international guidelines that can be used as a template for country-specific adaptions.

Surprisingly, although AK are a very common health problem especially in fair-skinned patients and account for large disease burden form a public health care perspective, we only identified three currently valid evidence- and consensus-based guidelines dealing with this topic, which were published within the last 5 years and matched our pre-defined eligibility criteria. The detailed international guideline on AK treatment developed by the International League of Dermatological Societies (ILDS) and published in 2015 was not included in this evaluation, as it had already expired in July 2018 [19].

Developing guidelines and keeping them up-do-date is labor- and cost-intensive. In the field of AK, developers are confronted with a vast number of treatment options including several monotherapies as well as combinations of them. Scanning all the evidence available is time-consuming and difficult. Besides, the quality of the body of evidence varies across interventions, making it difficult to compare the efficacy of different approaches and derive recommendations. However, regular updating is crucial for maintaining the quality of the provided guidance as seen in the case of ingenol mebutate (IMB), which was approved by the Food and Drug Administration (FDA) and European Medicines Agency (EMA) as a topical intervention for AK in 2012 [29]. Recently, the EMA decided to suspend its usage in January 2020, as a post-marketing surveillance study had shown that patients treated with IMB showed a higher incidence of skin cancer compared to imiquimod. Surprisingly, all evaluated guidelines recommended the use of IMB, but only the German guideline was amended in March 2020 in order to provide a footnote to no longer recommend the use of IMB [5]. This example underlines that continuous updating of guidelines is indispensable for state of the art patient care.

The German guideline achieved the highest scores in all domains of AGREE II and AGREE-REX ranging from 65% (applicability) to 92% (stakeholder involvement and editorial independence). On the other hand, the Canadian guideline was rated as the guideline with the poorest methodological quality in our appraisal with scores ranging from 31% (applicability) to 71% (clarity of presentation). Overall, the domain “clarity of presentation” was rated best among all evaluated domains ranging from 71% (Canadian guideline) to 91% (German guideline), indicating that the recommendations provided by the guidelines are unambiguous and clear and can be easily found in the guideline texts. In contrast, the domains “applicability” and “values and preferences” achieved the lowest scores ranging from 31 to 65% and 33 to 67%, respectively. Interestingly, these two domains also achieved only low scores in a recent appraisal of currently available evidence- and consensus-based melanoma guidelines [30], indicating that guideline developers may not pay sufficient attention to these domains in general and tend to neglect them. As the German evidence- and consensus-based guideline is being updated at the moment [10,25], it is of utmost importance to improve this weakness in the update.

Major differences between the guidelines were observed in the evaluation of the domain “stakeholder involvement”. Here, the German guideline achieved a good result of 92%, whereas both the UK and the Canadian guideline were rated worse, achieving only 37 and 41%, respectively. The lack of participation of important target groups may severely hamper the implementation of the recommendations into the real world setting. Thus, when updating these guidelines, developers should particularly focus on this part to improve the overall quality in the future. Especially patient representatives should ultimately be involved in the development of guidelines and might be actively approached through patient support groups.

Overall, the German guideline achieved the highest scores in all domains in both instruments. This might be due to the fact that the AWMF and DKG, which guide the process of oncological guideline development in Germany, provide not only support but also build a solid methodological framework of rules for the guideline authors they have to adhere to. Besides, the German guideline provided by far the most detailed Supplementary Materials including very detailed descriptions, which facilitated the identification of relevant content for the appraisal. According to the UK guideline [6], the AGREE II instrument also served as a guide for its development. This might explain why the UK guideline achieved better results compared to the Canadian guideline, although both guideline texts are similarly short.

This study has several limitations. We only evaluated the methodological quality of the guidelines, but not the content or the medical content of the recommendations themselves. This might be problematic as seen in the abovementioned case of IMB. Furthermore, we cannot fully exclude that the six reviewers may have been biased as all of them are from Germany. Additionally, three members of this research team (T.S., C.B., M.V.H.) were at least partly involved in the development process of the evaluated German guideline. However, these three were not part of the appraisal team and did not evaluate the quality of any guideline. Furthermore, the language restrictions to English and German may have led to the exclusion of relevant guidelines and may have introduced risk for selection bias.

## 5. Conclusions

Taken together, we identified three currently available guidelines on AK treatment that were published within the last five years. Two of them showed substantial methodological weaknesses. Only the German guideline, which was rated as the best in this evaluation, fulfilled most of the evaluated criteria and, therefore, may be used as a role model for developing or updating future guidelines. Paying special attention to the domains “applicability” and “values and preferences” that achieved low scores in all three guidelines is required.

## Data Availability

The data presented in this study are available on request from the corresponding author.

## Supplementary Materials

The following are available online at https://www.mdpi.com/1718-7729/28/1/93/s1, Table S1: Overview of the guideline databases searched for “aktinische keratose, actinic keratosis, actinic keratoses, solar keratoses, senile keratoses, field cancerization, precancerous lesions”, Table S2: Overview of the search strategy in Medline and Embase via Ovid.

## References

[1] Cantisani C., Paolino G., Melis M., Faina V., Romaniello F., Didona D., Cardone M., Calvieri S. (2016). Actinic Keratosis Pathogenesis Update and New Patents. Recent Pat. Inflamm Allergy Drug Discov..

[2] Moy R.L. (2000). Clinical presentation of actinic keratoses and squamous cell carcinoma. J. Am. Acad. Derm..

[3] Criscione V.D., Weinstock M.A., Naylor M.F., Luque C., Eide M.J., Bingham S.F., The Department of Veteran Affairs Topical Tretinoin Chemoprevention Trial Group (2009). Actinic keratoses: Natural history and risk of malignant transformation in the Veterans Affairs Topical Tretinoin Chemoprevention Trial. Cancer.

[4] Poulin Y., Lynde C.W., Barber K., Vender R., Claveau J., Bourcier M., Ashkenas J., Canadian Non-Melanoma Skin Cancer Guidelines Committee (2015). Non-melanoma Skin Cancer in Canada Chapter 3: Management of Actinic Keratoses. J. Cutan Med. Surg..

[5] (2020). Leitlinienprogramm Onkologie (Deutsche Krebsgesellschaft, D.K., AWMF), S3-Leitlinie Aktinische Keratose und Plattenepithelkarzinom der Haut, Langversion 1.1. https://www.leitlinienprogramm-onkologie.de/leitlinien/aktinische-keratosen-und-plattenepithelkarzinom-der-haut/.

[6] de Berker D., McGregor J.M., Mohd Mustapa M.F., Exton L.S., Hughes B.R. (2017). British Association of Dermatologists' guidelines for the care of patients with actinic keratosis 2017. Br. J. Derm..

[7] Del Duca E., Manfredini M., Petrini N., Farnetani F., Chester J., Bennardo L., Schipani G., Tamburi F., Sannino M., Cannarozzo G. (2019). Daylight Photodynamic Therapy with 5-aminolevulinic acid 5% gel for the treatment of mild-to-moderate inflammatory acne. G Ital. Derm. Venereol..

[8] Steeb T., Wessely A., Leiter U., French L.E., Berking C., Heppt M.V. (2020). The more the better? An appraisal of combination therapies for actinic keratosis. J. Eur. Acad. Derm. Venereol..

[9] Heppt M.V., Steeb T., Leiter U., Berking C. (2019). Efficacy of photodynamic therapy combined with topical interventions for the treatment of actinic keratosis: A meta-analysis. J. Eur. Acad. Derm. Venereol..

[10] Heppt M.V., Leiter U., Steeb T., Amaral T., Bauer A., Becker J.C., Breitbart E., Breuninger H., Diepgen T., Dirschka T. (2020). S3 guideline for actinic keratosis and cutaneous squamous cell carcinoma—Short version, part 1: Diagnosis, interventions for actinic keratoses, care structures and quality-of-care indicators. J. Dtsch. Derm. Ges..

[11] Heppt M.V., Steeb T., Ruzicka T., Berking C. (2019). Cryosurgery combined with topical interventions for actinic keratosis: A systematic review and meta-analysis. Br. J. Derm..

[12] Steeb T., Schlager J.G., Kohl C., Ruzicka T., Heppt M.V., Berking C. (2019). Laser-assisted photodynamic therapy for actinic keratosis: A systematic review and meta-analysis. J. Am. Acad. Derm..

[13] Steeb T., Wessely A., von Bubnoff D., Dirschka T., Drexler K., Falkenberg C., Hassel J.C., Hayani K., Huning S., Kahler K.C. (2020). Treatment Motivations and Expectations in Patients with Actinic Keratosis: A German-Wide Multicenter, Cross-Sectional Trial. J. Clin. Med..

[14] Brouwers M.C., Kho M.E., Browman G.P., Burgers J.S., Cluzeau F., Feder G., Fervers B., Graham I.D., Grimshaw J., Hanna S.E. (2010). AGREE II: Advancing guideline development, reporting and evaluation in health care. CMAJ.

[15] AGREE-REX Research Team The Appraisal of Guidelines Research & Evaluation—Recommendation EXcellence (AGREE-REX) [Electronic Version]. https://www.agreetrust.org/resource-centre/agree-rex-recommendation-excellence/.

[16] Steeb T., Hayani K.M., Forster P., Liegl R., Toussaint F., Schlaak M., Berking C., Heppt M.V. (2020). Guidelines for uveal melanoma: A critical appraisal of systematically identified guidelines using the AGREE II and AGREE-REX instrument. J. Cancer Res. Clin. Oncol..

[17] AGREE Next Steps Consortium The AGREE II Instrument [Electronic Version]. http://www.agreetrust.org.

[18] Landis J.R., Koch G.G. (1977). The measurement of observer agreement for categorical data. Biometrics.

[19] Werner R.N., Stockfleth E., Connolly S.M., Correia O., Erdmann R., Foley P., Gupta A.K., Jacobs A., Kerl H., Lim H.W. (2015). Evidence- and consensus-based (S3) Guidelines for the Treatment of Actinic Keratosis—International League of Dermatological Societies in cooperation with the European Dermatology Forum—Short version. J. Eur. Acad. Dermatol. Venereol..

[20] Werner R.N., Stockfleth E., Connolly S.M., Correia O., Erdmann R., Foley P., Gupta A.K., Jacobs A., Kerl H., Lim H.W. Evidence- and Consensus-Based (S3) Guidelines for the Treatment of Actinic Keratosis—International League of Dermatological Societies in Cooperation with the European Dermatology Forum—Long Version (Online Supplement). https://onlinelibrary.wiley.com/action/downloadSupplement?doi=10.1111%2Fjdv.13179&file=jdv13179-sup-0001-SuppInfo.pdf.

[21] Scottish Intercollegiate Guidelines Network (SIGN) Management of Primary Cutaneous Squamous Cell Carcinoma. https://www.sign.ac.uk/sign-140-management-of-primary-cutaneous-squamous-cell-carcinoma.

[22] Peris K., Calzavara-Pinton P.G., Neri L., Girolomoni G., Malara G., Parodi A., Piaserico S., Rossi R., Pellacani G. (2016). Italian expert consensus for the management of actinic keratosis in immunocompetent patients. J. Eur. Acad. Dermatol. Venereol..

[23] Baaten G., Buis P., Damen Z., De Haas E., Van der Heide W., Opstelten W., Smeink P., De Vijlder H. NHG-Standaard Verdachte huidafwijkingen. https://www.nhg.org/standaarden/samenvatting/verdachte-huidafwijkingen.

[24] Gutzmer R., Wiegand S., Kolbl O., Wermker K., Heppt M., Berking C. (2019). Actinic keratosis and cutaneous squamous cell carcinoma—Treatment options. Dtsch. Arztebl. Int..

[25] Leiter U., Heppt M.V., Steeb T., Amaral T., Bauer A., Becker J.C., Breitbart E., Breuninger H., Diepgen T., Dirschka T. (2020). S3 guideline for actinic keratosis and cutaneous squamous cell carcinoma (cSCC)—Short version, part 2: Epidemiology, surgical and systemic treatment of cSCC, follow-up, prevention and occupational disease. J. Dtsch. Derm. Ges..

[26] Bickers D.R., Lim H.W., Margolis D., Weinstock M.A., Goodman C., Faulkner E., Gould C., Gemmen E., Dall T., American Academy of Dermatology A. (2006). The burden of skin diseases: 2004 a joint project of the American Academy of Dermatology Association and the Society for Investigative Dermatology. J. Am. Acad. Derm..

[27] Werner R.N., Marinovic B., Rosumeck S., Strohal R., Haering N.S., Weberschock T., Dreher A.C., Nast A. (2016). The quality of European dermatological guidelines: Critical appraisal of the quality of EDF guidelines using the AGREE II instrument. J. Eur. Acad. Derm. Venereol..

[28] Nast A., Spuls P.H., Ormerod A.D., Reytan N., Saiag P.H., Smith C.H., Rzany B. (2009). A critical appraisal of evidence-based guidelines for the treatment of psoriasis vulgaris: ‘AGREE-ing’ on a common base for European evidence-based psoriasis treatment guidelines. J. Eur. Acad. Derm. Venereol..

[29] Gras J. (2013). Ingenol mebutate: A new option for actinic keratosis treatment. Drugs Today.

[30] Steeb T., Wessely A., Drexler K., Salzmann M., Toussaint F., Heinzerling L., Reinholz M., Berking C., Heppt M.V. (2020). The Quality of Practice Guidelines for Melanoma: A Methodologic Appraisal with the AGREE II and AGREE-REX Instruments. Cancers (Basel).

