# Supplementary material for: A Critical Appraisal of Evidence- and Consensus-Based Guidelines for Actinic Keratosis
Source: Curr Oncol. 2021 Feb 19;28(1):950–60. doi: 10.3390/curroncol28010093 (PMC7985770; doi:10.3390/curroncol28010093)
Supplement: Supplementary file 1 [file curroncol-28-00093-s001.pdf]

Current Oncology

Supplementary materials

A Critical Appraisal of Evidence- and Consensus-Based Guidelines for Actinic Keratosis

Anja Wessely [1,2], Theresa Steeb [1,2], Franz Heppt [1,2], Annkathrin Hornung [1,2], Matthias D. Kaufmann [1,2], Elias A.T. Koch [1,2], Frédéric Toussaint [1,2], Michael Erdmann [1,2], Carola Berking [1,2] and Markus V. Heppt [1,2,*]

Supplementary Table S1: Overview of the guideline databases searched for "aktinische keratose, actinic keratosis, actinic keratoses, solar keratoses, senile keratoses, field cancerization, precancerous lesions".

Guideline database	Initial hits
EADV homepage	3
Arbeitsgemeinschaft der Wissenschaftlichen Medizinischen Fachgesellschaften: AWMF (https://www.awmf.org/leitlinien/leitlinien-suche.html)	13
Ärztliches Zentrum für Qualität in der Medizin: ÄZQ (leitlinien.de)	0
Guidelines International Network: GIN (g-i-n.net)	3
Agency for Healthcare Research and Quality: NGC (guidelines.gov/)	11
Scottish Intercollegiate Guidelines Network: SIGN (sign.ac.uk/)	1
National Institute for Health and Care Excellence: NICE (guidance.nice.org.uk/CG/Published)	1
Arzneimittelkommission der deutschen Ärzteschaft (AkdÄ) http://www.akdae.de	21
Dutch Guidelines (oncoline.nl/index.php?language=en)	0
NCCN (https://www.nccn.org/)	0
Cross-references	16

Supplementary Table S2: Overview of the search strategy in Medline and Embase via Ovid.

**Search query in Medline (Ovid MEDLINE(R) and Epub Ahead of Print, In-Process & Other Non-Indexed Citations, Daily and Versions(R) 1946 to October 22, 2019; n = 959 hits)**
1. practice guideline.mp. or exp Practice Guideline/
2. health planning guideline.mp. or exp Health Planning Guidelines/
3. exp Guideline/ or guideline.mp. or exp Guideline Adherence/
4. guidance.mp.
5. evidence-based medicine.mp or exp Evidence-Based Medicine/
6. care pathway.mp.
7. consensus.mp. or exp Consensus Development Conference/ or exp Consensus Development Conferences, NIH as Topic/
8. 1 or 2 or 3 or 4 or 5 or 6 or 7
9. actinic keratosis.mp. or exp Keratosis, Actinic/
10. solar keratosis.mp.
11. senile keratosis.mp.
12. field change.mp.
13. actinically damaged field.mp.
14. exp Precancerous Conditions/ or field-cancerized.mp.
15. actinic keratosis.mp.
16. 9 or 10 or 11 or 12 or13 or 14 or 15
17. 8 and 16
**Search query in Embase (Embase 1974 to 2019 October 22 n=1,584 hits)**
1. actinic keratosis.mp. or actinic keratosis/
2. solar keratosis.mp.
3. senile keratosis.mp.
4. field change.mp.
5. actinically damaged field.mp.
6. field-cancerized.mp. or exp precancer/
7. actinic keratosis.mp.
8. 1 or 2 or 3 or 4 or 5 or 6 or 7
9. practice guideline.mp. or exp practice guideline/
10. health care planning.mp. or exp health care planning/
11. clinical pathway.mp. or exp clinical pathway/
12. guidance.mp.
13. exp consensus/ or consensus.mp.
14. decision making.mp. or exp decision making/
15. exp consensus/ or exp consensus development/ or consensus development.mp.
16. evidence-based medicine.mp. or exp evidence based medicine/ or medical decision making/
17. 8 and 16
